# Beneficial Effects of a Nutraceutical Combination on Lipid Profiles in Children with Moderate and Severe Hypercholesterolemia

**DOI:** 10.3390/biom14121608

**Published:** 2024-12-16

**Authors:** Anastasia Garoufi, Maria Papadaki, Michalis Kalogiannis, Urania Zerva, Marietta Charakida, Antonios Marmarinos, Achilleas Attilakos

**Affiliations:** 1 Lipid Outpatient Unit, 2nd Department of Pediatrics, Medical School, National and Kapodistrian University of Athens (NKUA), “P. & A. Kyriakou” Children’s Hospital, Thivon & Levadias Str., 11527 Athens, Greece; angaruf@med.uoa.gr (A.G.); papadaki.mairh@gmail.com (M.P.); mcharacida@med.uoa.gr (M.C.); 2 Third Department of Pediatrics, Medical School, National and Kapodistrian University of Athens (NKUA), “Attikon” General Hospital, 12462 Athens, Greece; mixalis_kalogiannis11@hotmail.com; 3 Nutrition Department, “P. & A. Kyriakou” Children’s Hospital, 11527 Athens, Greece; rzerva@gmail.com; 4 Laboratory of Clinical Biochemistry—Molecular Diagnostic, 2nd Department of Pediatrics, Medical School, National and Kapodistrian University of Athens (NKUA), “P. & A. Kyriakou” Children’s Hospital, 24 Mesogeion Avn, 11527 Athens, Greece; antmar@med.uoa.gr

**Keywords:** dyslipidemia, lipid profile, nutraceuticals, red yeast rice, phytosterols

## Abstract

The aim of the present study was to evaluate the efficacy and safety of the long-term use of a dietary supplement containing red yeast rice (RYR), combined with other natural compounds, in children and adolescents with primary hypercholesterolemia. A nutraceutical, containing RYR, policosanols, coenzyme Q10, astaxanthin and folic acid (commercial name: Armolipid), was administered once daily in 84 children/adolescents with moderate or severe primary hypercholesterolemia. Moreover, 19 of the participants consumed 1.5–2.5 g of phytosterols daily until the initiation of dietary supplementation with Armolipid. Clinical and laboratory evaluation took place before and 6 and 16 months after treatment. Nutraceutical consumption resulted in a significant decrease in total cholesterol, low-density lipoprotein cholesterol, non-high-density lipoprotein cholesterol and apolipoprotein B levels, which was maintained with long-term administration (*p* < 0.001). No changes were observed in high-density lipoprotein cholesterol, triglycerides, apolipoprotein A1 and lipoprotein (a) levels. In children previously on phytosterol supplementation, Armolipid use exerted a further significant reduction in atherogenic lipoproteins. Armolipid may be an effective and safe complementary treatment for children with moderate and severe hypercholesterolemia. More prospective studies on larger cohorts are needed to establish the role of nutraceuticals containing RYR, policosanols and other natural compounds in the treatment of children with hypercholesterolemia.

## 1. Introduction

Heterozygous familial hypercholesterolemia (HeFH) is a common genetic disease characterized by lifelong elevation of low-density lipoprotein cholesterol (LDL-C), which can lead to early-onset atherosclerosis and increased risk of cardiovascular disease (CVD) [1,2]. In recent decades, growing data have emphasized the importance of early detection and intervention in HeFH subjects, as research findings clearly demonstrate that the cumulative risk of exposure to elevated LDL-C levels from birth accelerates the progression of atherosclerosis. Thus, lowering cholesterol levels at a young age reduces the risk of CVD later in life [1,2,3].

Unfortunately, childhood HeFH is mostly underdiagnosed or undertreated [4,5]. Therapeutic lifestyle interventions, including dietary modifications, intense physical activity and promotion of healthy habits, constitute the most important strategy for managing dyslipidemia in children. A low-lipid diet in combination with the consumption of fruit, vegetables, whole grains, beans, fish and lean meats, such as Mediterranean-style diets, should be encouraged. Sedentary lifestyle and smoking should be strongly discouraged. In one large randomized controlled trial, evaluating the efficacy of dietary intervention on the lipid profile, an average LDL-C reduction of 11.8% was reported in children with medium–high hypercholesterolemia [6].

Recent data show that the majority of children affected by genetic dyslipidemias, even those under hypolipidemic drug therapy, do not reach the LDL-C lowering goals [1,2,3]. Functional foods or nutraceuticals (NCs) are natural compounds that can have a positive impact on human health for the prevention or therapy of a specific disease. Recently, lipid-lowering NCs, alone or in combination, are gaining a growing interest, as they seem to be an effective complementary therapy in adult patients with mild or modest hypercholesterolemia or with intolerance to conventional drug therapy. However, there are few studies evaluating the efficacy and safety of the NC combinations compared to that of each component they contain [7,8,9,10]. Notably, guidelines of the International Lipid Expert Panel as well as the European Society of Cardiology and the European Atherosclerosis Society (EAS) recommend the use of dietary supplements and a balanced diet to improve lipid profile [11,12].

Red yeast rice (RYR), a Chinese rice cultivated with the mold *Μonascus purpureus*, is currently the most well-studied, after soluble fibers and phytosterols, as an effective lipid-lowering nutraceutical [13]. Its main bioactive compound is a natural statin, the monacolin K, a 3-hydroxy-3-methyl-glutaryl-coenzyme A (HMG-CoA) reductase inhibitor, structurally and functionally similar to lovastatin. RYR efficacy, as well as its safety profile, is considered to be comparable to those of low-dose statins [9,13]. Data show that daily consumption of monacolin K leads to a reduction in low-density lipoprotein cholesterol (LDL-C) levels, up to 15–25% within 6 to 8 weeks. Furthermore, growing evidence support that RYR use improves the structure and function of vessels as well as inflammatory biomarkers [11,13]. Adverse effects are similar to those of statins but are rare when low doses of monacolin K are used. Moreover, many adverse effects have been attributed to citrinin, a substance produced during rice fermentation by *Μonascus purpureus*, in experimental models. Thus, the recommendation is to use citrinin-free RYR products [8,9,11].

As NCs have various mechanisms of lipid-lowering action, their combined use could have additive or synergistic effects. Recent data support the potential use of nutraceutical combinations, with the majority of them containing RYR combined with other substances such as policosanols, berberine, astaxanthin, coenzyme Q10 and folic acid [8,9]. Policosanols are long-chain aliphatic alcohols, which inhibit the action of HMG-CoA reductase and bile acid absorption, with the results of studies about its hypolipidemic action being conflicting. Berberine is a plant-derived alkaloid drug which improves LDL-C excretion by different mechanisms and has a beneficial effect on glucose metabolism and blood pressure [8]. Coenzyme Q10, an antioxidant and anti-inflammatory factor, inhibits LDL-C oxidation and may improve lipid profile. Astaxanthin has an antioxidant and anti-inflammatory action, and an effect on lipid metabolism, although its supplementation had no effect on total cholesterol levels in adult patients [8,9,11]. Finally, folic acid administration resulted in a significant reduction in total cholesterol (TC) and LDL-C levels in adults with atherosclerotic risk factors [14].

The majority of clinical evidence evaluating efficacy and safety of NCs involves adult dyslipidemic patients [8]. In the latest EAS guidelines, NCs’ use has also been indicated for children, older than 6 years, with familial hypercholesterolemia [15]. There are few studies concerning the administration of NCs in children and adolescents with dyslipidemia. Most of them refer to the administration of soluble fibers and phytosterols to a limited number of subjects, while only one study refers to the early effect of supplementation with RYR combined with policosanols on lipid profile in childhood [16,17].

As existing data for RYR use in childhood are extremely limited, the aim of the present study was to evaluate the efficacy and safety of the long-term consumption of a dietary supplements containing RYR, combined with other NCs, in children and adolescents with primary hypercholesterolemia.

## 2. Material and Methods

### 2.1. Study Design

A prospective single-center cohort study was conducted at Outpatient Lipid Unit of 2nd Department of Pediatrics of the National and Kapodistrian University of Athens at the “Panagiotis & Aglaia Kyriakou” Children’s Hospital in Greece. The study complies with the Declaration of Helsinki, and the protocol was approved by the “Panagiotis & Aglaia Kyriakou” Children’s Hospital ethical committee (approval number: 1474/10 January 2020). The study has been registered in ClinicalTrials.gov (NCT06045377). Informed parental consent was obtained prior to study enrollment.

### 2.2. Study Population

Ninety children and adolescents, aged 7 to 16 years, with primary dyslipidemia were enrolled in the study. The cohort enrolment criteria were age ≥ 7 years and LDL-C > 150 mg/dL in more than two measurements, after therapeutic lifestyle changes for at least 6 months. The exclusion criteria were secondary hypercholesterolemia, presence of chronic disease or growth/developmental disorder, abnormal liver, kidney or thyroid function and prior use of hypolipidemic or other medication, at least 6 months before participation in the study.

All participants had a family history for hypercholesterolemia (LDL-C ≥ 95th percentile), and most of them had a family history for premature CVD in first- or/and second-degree relatives. Eleven of them had a genetically confirmed diagnosis of HeFH, while the rest had a probable or positive HeFH according to the Dutch Lipid Clinic Network criteria [18]. None of them had hypertriglyceridemia, even those with increased body mass index (BMI).

All children followed a low-cholesterol diet as directed by a trained dietician and had moderate or intense physical activity, for at least 6 months before participation in the study. In addition, during the last 6 months, 19 of them agreed to consume 1.5–2.5 g of plant sterols daily in the form of a yogurt drink or spread. Lifestyle and eating habits did not change throughout the study.

A nutraceutical named Armolipid (Rottapharm S.p.A., Monza, Italia), containing five natural substances formulated as a tablet, was recommended in all participants. Every tablet contained 200 mg red yeast rice (RYR) extract equivalent to 3 mg of monacolin K, 10 mg policosanols, 0.2 mg folic acid, 2.0 mg coenzyme Q10 and 0.5 mg astaxanthin and was citrinin-free. It was administered once daily with lunch.

Participants and their parents were interviewed using a form created specifically for this research. Details concerning compliance to supplement intake, as well as possible adverse effects, were recorded and analyzed. A total of 6 out of 90 children were excluded from the study because they did not comply with the recommendation for taking the supplement (compliance rate 93.3%). The remaining 84 children had one evaluation, and 64 of them agreed to have a second re-evaluation under Armolipid treatment.

### 2.3. Clinical and Laboratory Evaluation

A clinical and laboratory evaluation of all children took place right before (Time 0, T_0_) and once (*n* = 84, Time 1, T_1_) or twice (*n* = 64, T_1_ and Time 2, T_2_) after the initiation of Armolipid.

The height (H) in cm and the body weight (BW) in kg were measured to the nearest 0.1 kg and 0.5 cm, respectively (TANITA, Corporation Tokyo, Japan), with children lightly dressed and barefoot. BMI was calculated as BW in kg per H in m^2^. The BW, H and BMI standard deviation scores (z-scores) were also calculated according to an age- and sex-specific calculator. Waist circumference (WC) in cm was measured, and the ratio of WC/height was calculated. Blood pressure, systolic (SBP) and diastolic (DBP) in mmHg, was measured three consecutive times using an automated oscillometric device (Dinamap V100, (GE Healthcare, Tampa, FL, USA), and the average value of the three measurements was used in the statistical analysis. Puberty stage was recorded according to Tanner stages (I–V) for girls and boys [19].

Total cholesterol (TC), LDL-C, high-density lipoprotein cholesterol (HDL-C), non-high-density lipoprotein cholesterol (non-HDL-C), triglycerides (TGs), apolipoprotein A1 (Apo-A1), apolipoprotein B (Apo-B), and lipoprotein (a) [Lp(a)] levels were evaluated in blood, after an overnight fast. Serum creatinine, glucose, aspartate and alanine aminotransferases (AST and ALT), creatine kinase (CK), and thyroid-stimulating hormone (TSH) were also measured. A full blood count was performed in all study participants.

TC, HDL-C, LDL-C, TGs, glucose, ALT, AST, CK and creatinine were measured on an automatic analyzer (Cobass Integra 800), using an enzymatic method (Roche Diagnostics, GmbH, Mannheim, Germany). Apo-A1, Apo-B, and Lp(a) were measured by an immunonephelometric assay (), with an intra-assay and inter-assay variation < 5% for all tests. Non-HDL-C was calculated as TC minus HDL-C. All lipid values are expressed in mg/dL. Hematological parameters (full blood count) were analyzed using the automated hematology analyzer SysmexXE-2100 (Roche Diagnostics). Manufacturers’ instructions were followed for all instruments.

All the children were healthy, and none of them had any infection in the two weeks preceding the check-up.

The primary outcome was the reduction in LDL-C serum levels. Secondary outcomes included improvement in other lipid parameters and the evaluation of possible adverse effects such as gastrointestinal symptoms or hepatic and muscle enzyme elevation.

### 2.4. Statistical Analysis

Qualitative data are presented with absolute and relative frequencies (%). Depending on the distribution (normal or not), quantitative data are presented with mean ± standard deviation or Median (Q25, Q75). Regularity was checked using the Shapiro–Wilk criterion, and graphically with the use of histograms, normal Q-Q plots and boxplots. To evaluate changes in lipid profile before and after Armolipid consumption in the total study population, a Friedman test was used (because of non-normality of the data). An individual Wilcoxon Signed Rank Test was conducted (using a Bonferroni adjusted alpha value) to control for Type 1 error. A one-way repeated measures ANOVA was conducted to evaluate changes in lipid profile after Armolipid supplementation in participants with no prior consumption of phytosterols (because of normality of the data in this group of children). Mixed between–within subjects’ analysis of variance was used for the comparison of Armolipid effect on the lipid profile of population subgroups. The impact of Tanner alteration on lipid profile improvement was examined by using a Mann–Whitney U Test, while a paired-samples *t*-test was conducted to evaluate differences in lipid levels before and after phytosterols consumption. Statistical analysis was performed with the statistical package SPSS, Version 25 (IBM SPSS Statistics for Windows, Armonk, NY, USA: IBM Corp.) and a probability value of *p* < 0.05 was considered statistically significant.

## 3. Results

### 3.1. Descriptive Characteristics of the Study Population

Six out of the ninety children and adolescents who were recommended Armolipid supplementation daily were excluded from the study because of bad compliance. Finally, 84 Caucasian children and adolescents, 41 (48.8%) males and 43 (51.2%) females, aged 7–16 years old (mean age: 9.9 years, ±2.1) were included in statistical analyses. Nineteen of the participants (22.6%) were consuming phytosterols, 1.5–2.5 g daily, at least 3 months before the enrollment in the study. The consumption was discontinued from the day of their inclusion to the study.

All 84 participants were clinically and biochemically evaluated once (T_1_), and 64 (76.2%) participants twice (T_1_ and T_2_), under Armolipid treatment. The median intervals between the baseline (T_0_) and 1st (T_1_) and 2nd (T_2_) evaluation were 6 (Q25–Q75: 5–8) and 16 (Q25–Q75: 11–19.7) months, respectively. The mean age of participants in T_0_, T_1_ and T_2_ was 9.9 (SD:2.0), 10.5 (SD:2.0) and 11.2 (SD:2.0) years, respectively. At baseline (T_0_), 47 (56%) of subjects were in pre-pubertal (Tanner stage 1) and 37 (44%) in pubertal stage (Tanner stages 2–5). At the T_1_ evaluation, 47.6% were in pre-puberty and 52.4% in puberty, while at the T_2_ evaluation, the respective percentages were 39% and 61%.

The clinical and biochemical variables of the 64 participants who were evaluated twice under Armolipid treatment are shown in Table 1.

### 3.2. Changes in Lipid Profile After Armolipid Supplementation in Total Study Population

A significant reduction in TC, LDL-C, non-HDL-C and Apo-B levels was observed after Armolipid treatment (*p* < 0.001), while HDL-C, Apo-A1 and TGs levels did not change significantly (Table 1). The absolute (mg/dL) and the percentage changes in lipids, lipoproteins and apolipoproteins levels after Armolipid consumption are presented in Table 2 (the changes in variables conform to normal distribution, so they are expressed as Mean (SD)).

At 1st evaluation after Armolipid administration (T_1_), a decrease in LDL-C, non-HDL-C and Apo-B ≥ 10% was observed in 60.7%, 63.1% and 66.7% of 84 participants. At the 2nd evaluation (T_2_, *n* = 64), the respective percentages were 78.1%, 70.3% and 59.4%. Moreover, a decrease in LDL-C, non-HDL-C and Apo-B from 5% to <10% from baseline levels was found in 14.3%, 11.9% and 7.1% of participants in T_1_ and in 6.3%, 14.1% and 12.5% in T_2_ evaluation.

None of the participants presented any gastrointestinal symptom or any liver, muscle or renal enzymes elevations during the study period (Table 1).

### 3.3. Changes in Lipid Profile After Armolipid Supplementation in Participants with No Prior Consumption of Phytosterols

**Figure 1:**
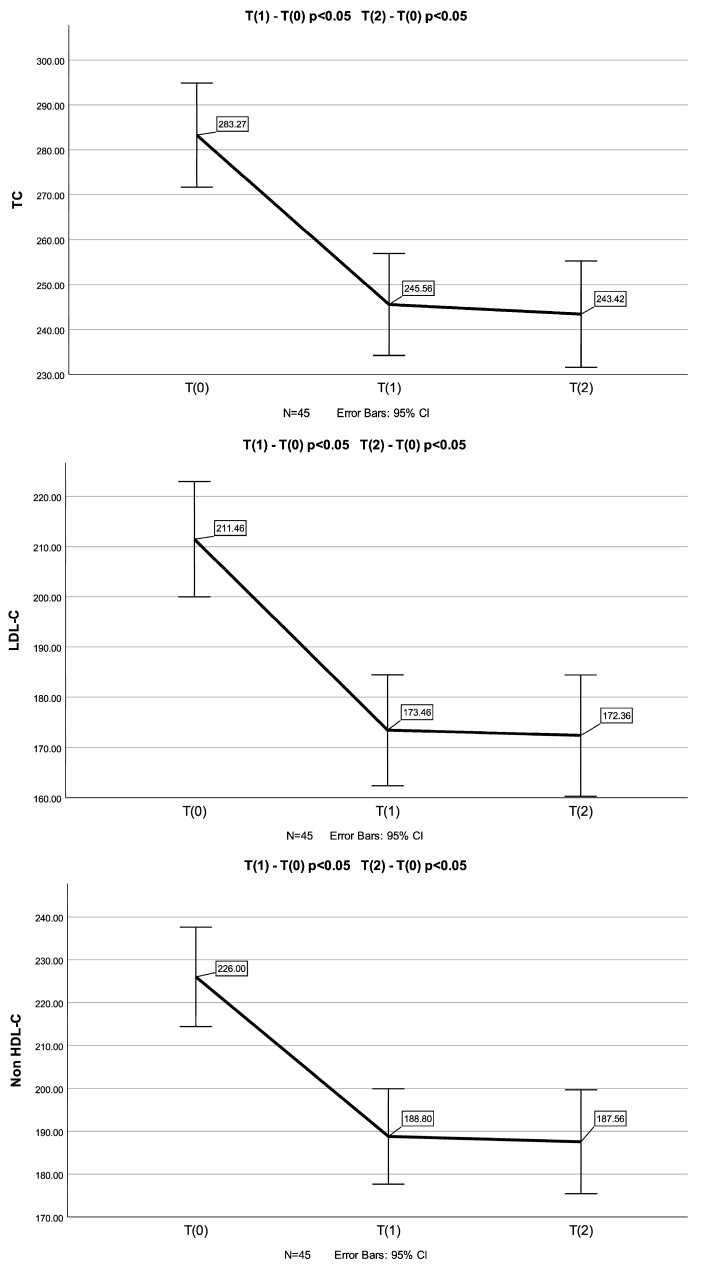

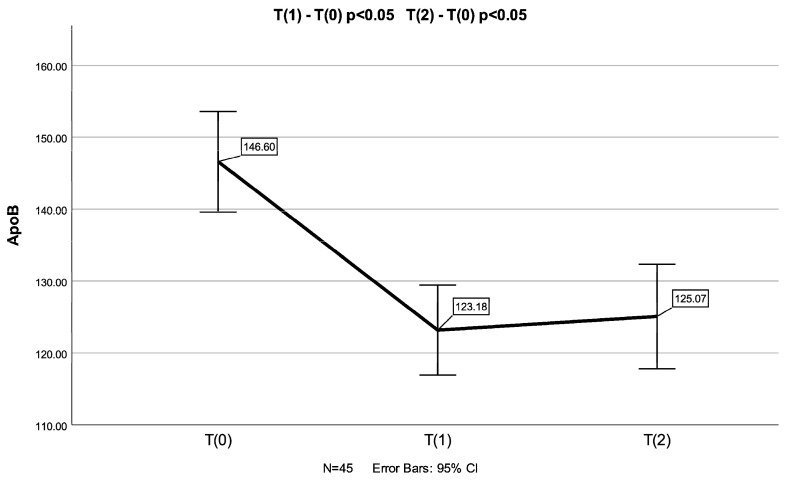
Changes in TC, LDL-C, non-HDL-C and Apo-B levels (mean values) in T_0_, T_1_ and T_2_ measurements in participants with no prior consumption of phytosterols (*n* = 45). Error bars represent standard deviations. *p* values were calculated with the one-way repeated measures ANOVA.

In the group of children who had not previously consumed plant sterols (*n* = 64), a statistically significant reduction in TC (*p* < 0.001), LDL-C (*p* < 0.001), non-HDL-C (*p* < 0.001) and Apo-B (*p* < 0.001) levels was found in T_1_ and T_2_ (*n* = 45) measurements compared to the baseline (T_0_) (a one-way repeated measures ANOVA was conducted because of normality of the data in this group of children). Appropriate post hoc tests (pairwise comparisons) revealed a significant decrease in TC (*p* < 0.001 and *p* < 0.001), LDL-C (*p* < 0.001 and *p* < 0.001), non-HDL-C (*p* < 0.001 and *p* < 0.001) and Apo-B (*p* < 0.001 and *p* < 0.001) levels between T_0_ and T_1,_ as well as between T_0_ and T_2_ measurements (Figure 1). There was no significant difference in TC, LDL-C, non-HDL-C and Apo-B levels between T_1_ and T_2_ measurements.

A one-way repeated measures ANOVA was conducted to compare scores on TC, LDL-C, non-HDL-C and Apo-B in three time periods. There was a significant effect for time (*p* < 0.001). Post hoc comparisons test, for all four parameters, indicated that the mean score for T(0) was significantly different from T(1) and from T(2). Also, the mean score for T(1) did not differ significantly from T(2).

No significant changes in HDL-C, TGs, Apo-A1 and Lp(a) levels were observed after the supplementation with Armolipid (T_0_ vs. T_1_ and T_2_).

No significant differences were found between BMI z-score, WC/H ratio, other biochemical parameters and TSH levels in times 0, 1 and 2. On the contrary, SBP, DBP and creatinine levels were significantly higher at T_1_ and T_2_ evaluation when compared with T_0_ (*p* < 0.001, age statistically significant, with a beta value for SBP, DBP and creatinine = 4.13, *p* < 0.001, =2.95, *p* < 0.001 and =0.03, *p* = 0.001, respectively). The alterations in Tanner stage during the study had no significant effect on the reduction in LDL-C, non–HDL-C and Apo-B levels.

### 3.4. Changes in Lipid Profile After Armolipid Supplementation in Participants with Prior Consumption of Phytosterols

**Figure 2:**
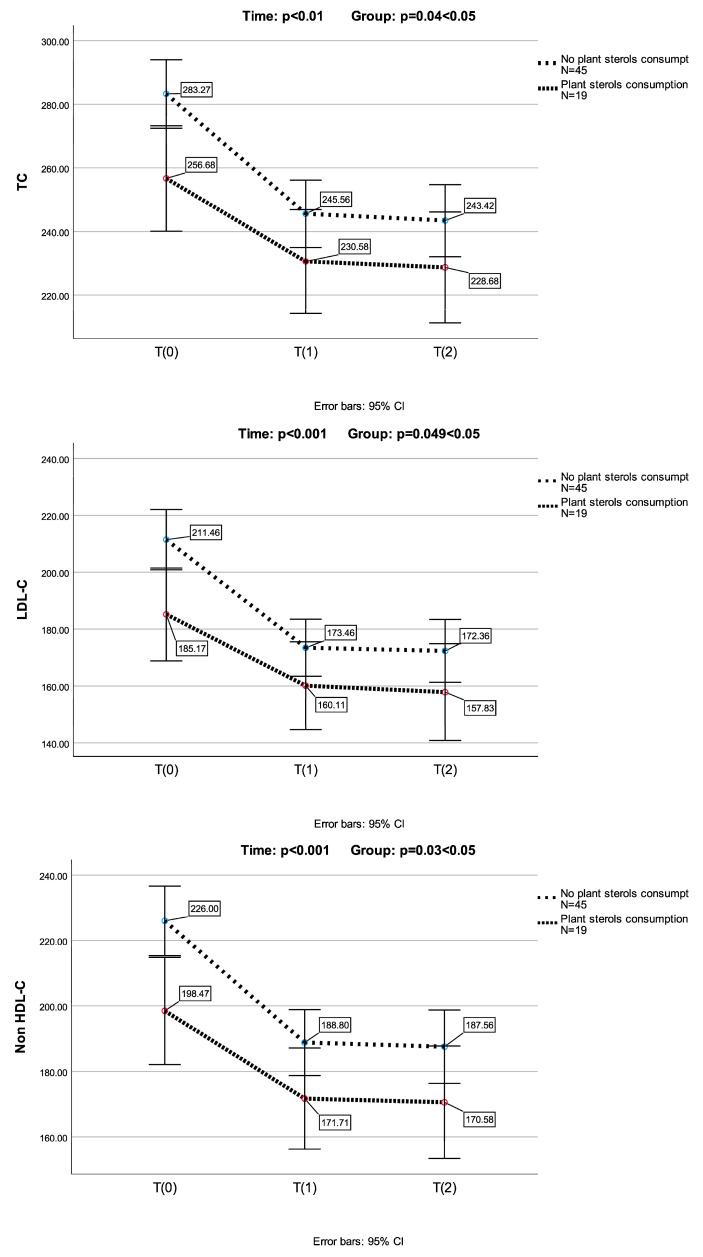

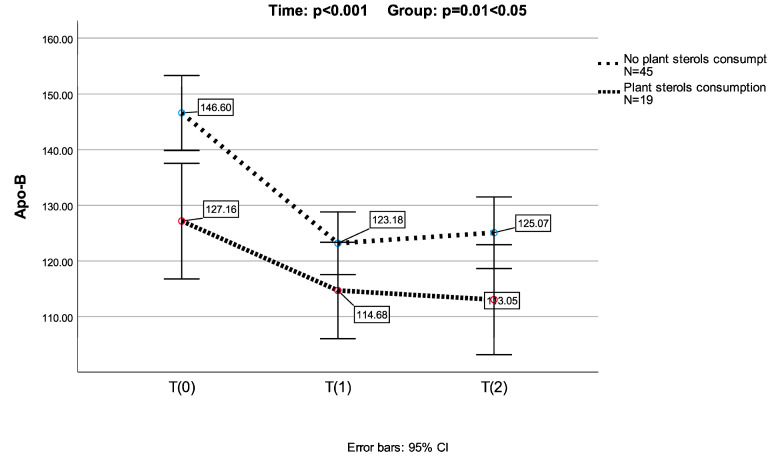
Armolipid’s lowering effect on TC, LDL-C, non-HDL-C and Apo-B levels (mean values) in population subgroups (no plant sterols consumption, *n* = 45; and plant sterols consumption, *n* = 19). Error bars represent standard deviations. *p* values were calculated with a mixed between–within subjects analysis of variance.

In the subgroup of children (*n* = 19), who had previously received plant sterols, previous supplementation with sterols resulted in a significant reduction in LDL-C levels (*p* < 0.001) (a one-way repeated measures ANOVA was conducted (because of normality of the data in this group of children)). In this group of children, TC, LDL-C, non-HDL-C and Apo-B further decreased significantly after the substitution of plant sterols by Armolipid (Figure 2). None of the above parameters presented any further reduction in the 2nd measurement.

A one-way repeated measures ANOVA was conducted to compare scores on TC, LDL-C, non-HDL-C and Apo-B in three time periods. There was a significant effect for time (*p* < 0.001). Post hoc comparisons test, for all four parameters, indicated that the mean score for T(0) was significantly different from T(1) and from T(2). Also, the mean score for T(1) did not differ significantly from T(2).

A mixed between–within subjects analysis of variance was conducted to assess the impact of plant sterols consumption (no plant sterols consumption group and plant sterols consumption group) on participants’ scores for TC, LDL-C, non-HDL-C and Apo-B across three time periods. Comparing the two groups, with and without prior plant sterols consumption, a significant difference in TC, LDL-C, non-HDL-C and Apo-B reduction was observed (*p* = 0.04, *p* = 0.049, *p* = 0.03 and *p* = 0.01, respectively) (Figure 2).

## 4. Discussion

In the present study, the long-term daily administration of 200 mg RYR extract equivalent to 3 mg of monacolin K, 10 mg policosanols, 0.2 mg folic acid, 2.0 mg coenzyme Q10 and 0.5 mg astaxanthin (commercial name: Armolipid) resulted in a significant long-lasting decrease in TC, LDL-C, non-HDL-C and Apo-B levels, in children with moderate and severe primary hypercholesterolemia. Almost 75% of participants had a reduction in atherogenic lipoproteins equal or higher than 5%, with more than 60% presenting a decrease equal to or above 10%. In contrast, Armolipid consumption did not have any significant effect on the levels of HDL-C, TGs, Apo-A1 and Lp(a). Moreover, in children who had previously consumed phytosterols, its substitution by Armolipid resulted in a further significant decrease. In the total population, the reduction in atherogenic lipoproteins was independent of treatment duration or baseline lipid levels. Finally, no adverse effects of Armolipid were observed.

To our knowledge, there is only one study evaluating the early effect of Armolipid on the lipid profile in children with dyslipidemia. In that study, similarly to our results, daily consumption of Armolipid for 4 weeks significantly reduced TC by 18.5%, LDL-C by 25.1% and Apo-B by 25.3%, compared to placebo. No significant differences were observed in HDL-C and Apo-A1 levels. Furthermore, contrary to our results, a significant reduction in TGs levels was also reported [17].

In adults, there is sufficient evidence showing the beneficial effect of RYR supplementation in the improvement of the lipid profile in patients with hypercholesterolemia [13]. A recent meta-analysis of 15 high-quality RCTs, including 1012 participants, showed that daily consumption of RYR in a dose of 200–4800 mg could be an effective and safe alternative for the treatment of dyslipidemic patients. RYR was effective in reducing TC, TG, LDL-C, apo-B and increasing HDL-C, and showed a synergistic action with other NCs [20].

Furthermore, growing evidence supports the association of RYR consumption, alone or in combination with other natural compounds, with the improvement of inflammatory biomarkers, endothelial function and arterial stiffness in adult dyslipidemic patients, as well as with the reduction in CVD risk in adults with previous myocardial infraction [8,10]. According to a recent meta-analysis, RYR consumption significantly reduced the risk of fatal and nonfatal cardiovascular events in patients with borderline hypercholesterolemia who were already affected by coronary artery disease [21].

The most well-studied combination of lipid-lowering NCs in adults is that of RYR, polycosanols and berberine (commercial name: Armolipid Plus). According to a recent meta-analysis of 12 studies, including 1050 subjects, the supplementation of the above combination had a significant effect on the improvement of BMI, lipid profile and inflammatory markers, which is consistent with improved cardiometabolic health [22]. The study of Galletti et al., in 158 adult patients with metabolic syndrome, showed a reduction in atherogenic lipoproteins, including small-dense LDL-C (sdLDL-C), after Armolipid Plus supplementation when compared with placebo [23]. Administration of Armolipid Plus in patients with familial combined hyperlipidemia had a similar effect on sdLDL-C levels, which suggests a potential role in atherosclerotic risk reduction [24]. However, there are no studies on the effect of RYR alone on sdLDL-C levels [25].

RYR, which inhibits HMG-CoA reductase enzyme, may have an additive or synergistic effect when combined with other NCs such as policosanols and astaxanthin. Policosanols inhibit cholesterol synthesis through deactivation of HMG-CoA reductase and activation of 5′ adenosine monophosphate-activated protein kinase (AMPK). Moreover, policosanols enhance the excretion of cholesterol and bile acids and increase the LDL-C uptake by LDL receptors [7,8,9,10,17]. Astaxanthin possesses antioxidant action, decreases biomarkers of oxidative stress, reduces inflammatory cytokines and modulates immune system [7,8,9,10,11].

Other natural compounds, such as coenzyme Q10 and folic acid, in combination with RYR, also had a beneficial effect on the lipid profile [14,26]. Moreover, an improvement in endothelial function and arterial stiffness, after the consumption of RYR combined with coenzyme Q10 for six months, has been reported in adults with moderate hypercholesterolemia [27]. Mechanisms by which coenzyme Q10 supplementation may improve lipid profile include downregulation of the lectin-like oxidized LDL receptor, reduction in reactive oxygen species and stimulation of AMPK. In addition, coenzyme Q10 improves HDL-mediated cholesterol efflux capacity, reduces the oxidation of circulating lipids, inhibits differentiation-induced adipogenesis and increases TG lipolysis [28]. Folic acid supplementation may increase HDL-C and decrease LDL-C by improving hyperomocysteinemia-associated hypomethylation which may cause lipid accumulation in tissues, inhibiting substances involved in HDL-particle assembly and regulating phosphatidylcholine, a phospholipid required for very low-density lipoprotein (VLDL) assembly and homeostasis. In addition, folic acid supplementation may improve endothelial function and prevent LDL oxidation [29].

In the present study, in children in whom prior consumption of plant sterols was replaced by Armolipid, a further significant reduction in LDL-C and other atherogenic lipoproteins levels, similar to that of children with no prior intake of phytosterols, was observed. In most of these children, the plant sterols supplementation had already resulted in a significant decrease in LDL-C, non-HDL-C and Apo-B levels. These results may indicate a stronger hypolipidemic effect of Armolipid compared to plant sterols. Given the fact that plant sterols inhibit cholesterol absorption in the intestines, a mechanism different than that of RYR, their combined use could have a synergistic effect in the improvement of lipid profile [8,9,11]. In the study of Cicero et al., including 90 adults with hypercholesterolemia, the daily administration of 800 mg phytosterols for 2 months had no effects on lipid profile. In contrast, the use of RYR extract, equivalent with 5 mg monacolin K, for 2 months had a beneficial effect on lipids, while an additive hypolipidemic effect with the simultaneous consumption of phytosterols and RYR for 2 months was observed [30]. Our study neither directly compared Armolipid to plant sterols nor did it evaluate any possible additive effect, since phytosterols supplementation was stopped when children started Armolipid.

Results of studies evaluating a possible anti-hypertensive effect of RYR are controversial [31,32]. In our study, SBP and DBP were significantly higher at T_1_ and T_2_ evaluation when compared with T_0_, possibly due to the older age of the participants.

Regarding the safety of RYR, a substance that mirrors statin effects, it is shown that it is similar to that of low-dose statins [11]. The use of citrinin-free RYP products minimizes the risk of adverse effects. In a recent study, a nutrivigilance-derived data analysis showed that adverse effects from the consumption of products containing RYR are very rare (0.037% of consumers), with only 0.0003% of consumers having serious adverse effects [33]. In the only short-term study in children, daily consumption of citrinin-free Armolipid had no serious adverse events [17]. Our findings are consistent with these data, as the long-term consumption of Armolipid was well tolerated and had no adverse clinical or biochemical effects. Combination of NCs with different actions by using low doses of each compound may reduce possible side effects.

This study has some limitations. First, there is no control group; thus, it is difficult to definitively attribute the changes in lipid profile only to Armolipid use. Secondly, the relatively small sample size, particularly within the subgroup with prior phytosterol consumption (*n* = 19), raises concerns about the generalizability of our findings. Thirdly, the limited sample size did not allow us to further examine possible effects of confounders such as age or pubertal stage on lipid profile. In addition, physical activity habits were not recorded, and the evaluation of participants’ compliance both with dietary instructions or Armolipid intake was based on information given by the participants as well as their parents. Finally, we did not include a group of children taking phytosterols and Armolipid simultaneously in order to explore potential synergistic effects.

The advantages of the present report are the prospective design, the long-term administration of the nutraceutical, the homogeneity of the study group and the inclusion of children not only with moderate but also with severe dyslipidemia (LDL-C > 190 mg/dL).

## 5. Conclusions

In conclusion, the present study shows a possible positive long-term effect and safety of a nutraceutical containing RYR, polycosanols, coenzyme Q10, astaxanthin and folic acid (Armolipid) in children with primary hypercholesterolemia, supporting its complementary use in those with moderate and severe hypercholesterolemia as well as in children with statin intolerance. The present results need to be confirmed by more prospective studies on larger cohorts to establish the role of NCs containing RYR, polycosanols and other natural compounds alone or in combination with plant sterols, in the treatment of children with hypercholesterolemia.

## Figures and Tables

**Table 1:** Clinical and biochemical variables of the 64 participants evaluated twice under Armolipid treatment.

	T_0_ (*n* = 64)	T_1_ (*n* = 64)	T_2_ (*n* = 64)	
	Median (Q25–Q75) ^a^	Median (Q25–Q75)	Median (Q25–Q75)	X^2^ (*p*-Value) ^b^
**BMI Z-score**	0.44 (−0.11/1.21)	0.44 (−0.29/1.11)	0.69 (−0.22/1.13)	1.004 (0.605)
**WC/H**	0.44 (0.41/0.47)	0.44 (0.42/0.47)	0.44 (0.40/0.47)	1.843 (0.398)
**SBP (mmHg)**	103.00 (98.25/110.00)	107.50 (100.25/114.50)	110.00 (105.00/118.75)	19.527 **(<0.001)**
**DBP (mmHg)**	61.00 (55.25/65.75)	60.00 (57.00/66.50)	63.00 (60.00/70.00)	22.842 **(<0.001)**
**TC (mg/dL)**	271.50 (243.75/301.00)	236.50 (212.25/262.75)	230.00 (212.50/265.75)	68.843 **(<0.001)**
**LDL-C (mg/dL)**	194.80 (173.50/227.25)	169.70 (144.25/183.60)	160.80 (143.55/192.35)	68.469 **(<0.001)**
**HDL-C (mg/dL)**	56.50 (48.00/64.00)	55.00 (49.00/62.00)	55.00 (49.00/62.75)	0.914 (0.633)
**Non-HDL-C (mg/dL)**	210.50 (184.42/248.75)	185.50 (158.00/196.00)	174.50 (156.25/204.50)	70.571 **(<0.001)**
**TGs (mg/dL)**	69.50 (57.50/79.75)	61.00 (51.00/90.87)	66.50 (53.25/88.25)	0.885 (0.642)
**Apo-A1 (mg/dL)**	140.50 (130.75/152.00)	141.00 (129.25/155.75)	140.00 (130.25/154.50)	1.266 (0.531)
**Apo-B (mg/dL)**	135.00 (124.25/155.00)	117.00 (108.25/131.00)	119.00 (107.25/130.00)	53.088 **(<0.001)**
**Lp(a) (mg/dL)**	9.10 (3.92/19.60)	9.50 (3.00/19.40)	9.00 (3.60/21.05)	2.846 (0.241)
**TSH (mlU/I)**	2.46 (1.88/3.19)	2.40 (1.88/3.13)	2.30 (1.67/3.20)	1.615 (0.446)
**Creatinine (mg/dL)**	0.50 (0.42/0.60)	0.50 (0.50/0.60)	0.58 (0.50/0.60)	18.506 **(<0.001)**
**AST (U/L)**	23.00 (20.00/26.00)	23.00 (20.00/26.00)	22.00 (18.25/25.00)	4.303 (0.116)
**ALT (U/L)**	16.00 (13.00/19.00)	16.00 (14.00/20.75)	16.50 (13.00/22.50)	0.678 (0.712)
**CK (U/L)**	108.00 (80.75/130.25)	106.50 (77.75/138.00)	115.00 (73.50/133.50)	1.170 (0.557)
**Glucose (mg/dL)**	84.50 (78.25/88.00)	87.00 (82.00/89.00)	87.00 (82.00/91.00)	3.857 (0.145)

^a^ Because of non-conformity of the data to normal distribution, they are expressed as Median (Q25–Q75). ^b^ Comparisons in scores across the three time points. X^2^ (*p*-Value) was calculated with the Friedman test because of non-conformity of the data to normal distribution. T_0_: Time 0 (Baseline), T_1_: Time 1, 1st evaluation under Armolipid treatment, T_2_: Time 2, 2nd evaluation under Armolipid treatment, BMI: body mass index, WC/H: waist circumference/height, SBP: Systolic Blood Pressure, DBP: Diastolic Blood Pressure, TC: total cholesterol, LDL-C: low-density lipoprotein cholesterol, HDL-C: high-density lipoprotein cholesterol, Non-HDL-C: non-high-density lipoprotein cholesterol, TGs: triglycerides, Apo-A1: apolipoprotein A1, Apo-B: apolipoprotein B, Lp(a): lipoprotein (a), TSH: thyroid-stimulating hormone, AST: aspartate aminotransferase, ALT: alanine aminotransferase, CK: creatine kinase.

**Table 2:** Absolute (mg/dL) and percentage (%) changes in lipid profile after Armolipid supplementation.

	T_1_ vs. T_0_ (*n* = 64) Mean (SD) ^a^	T_2_ vs. T_0_ (*n* = 64) Mean (SD)
**TC decrease (mg/dL)**	34.26 (27.35)	36.33 (27.06)
**TC decrease (%)**	12.07 (9.23)	12.95 (9.36)
**LDL-C decrease (mg/dL)**	34.16 (24.14)	35.61 (23.43)
**LDL-C decrease (%)**	16.30 (10.69)	17.35 (10.96)
**Non-HDL-C decrease (mg/dL)**	34.10 (25.16)	35.31 (23.52)
**Non-HDL-C decrease (%)**	15.18 (10.44)	16.10 (10.22)
**Apo-B decrease (mg/dL)**	20.17 (18.42)	19.32 (18.81)
**Apo-B decrease (%)**	13.36 (11.61)	12.95 (12.64)

^a^ Because of conformity of the data to normal distribution they are expressed as Mean (SD). T_0_: Time 0 (Baseline), T_1_: Time 1, 1st evaluation under Armolipid treatment, T_2_: Time 2, 2nd evaluation under Armolipid treatment, TC: total cholesterol, LDL-C: low-density lipoprotein cholesterol, Non-HDL-C: non-high-density lipoprotein cholesterol, Apo-B: apolipoprotein B.

## Data Availability

The original contributions presented in the study are included in the article, further inquiries can be directed to the corresponding author.
